# A Diagnostic Dilemma Upon Discovery of a Left Ventricle Mass: A Case Report

**DOI:** 10.1002/ccr3.70553

**Published:** 2025-06-18

**Authors:** Hedieh Alimi, Ali Tajik

**Affiliations:** ^1^ Vascular and Endovascular Surgery Research Center, Faculty of Medicine Mashhad University of Medical Sciences Mashhad Iran; ^2^ Student Research Committee, Faculty of Medicine Mashhad University of Medical Sciences Mashhad Iran

**Keywords:** cardiac tumor, echocardiography, left ventricular mass, thrombus

## Abstract

Left ventricular mass is an uncommon condition, with thrombus being the most frequent cause. In this report, we present a complicated case of a cardiac mass (a left ventricular mass) and provide educational insights on distinguishing between cardiac tumors and left ventricular thrombus, using imaging modalities like echocardiography.

**Abbreviations**
A4C: apical four chamber
CLL: chronic lymphocytic lymphoma
LV: left ventricular
MI: myocardial infarction
TTE: transthoracic echocardiography

**Summary**

- The management of patients with cardiac masses presents significant challenges.
- Making accurate clinical judgments requires a thorough assessment of the patient's history and physical examination, alongside the appropriate use of paraclinical tools to establish the correct diagnosis.

## 1 Introduction

Left ventricular mass is an uncommon condition, with thrombus being the most prevalent cause [1]. Echocardiography is a highly valuable method of examination for assessing the mass of the left ventricle. We are reporting a case with an underlying malignancy presenting with dyspnea and incidental left ventricular mass on transthoracic echocardiography (TTE).

## 2 History of Presentation: How the Patient Was Admitted, Physical Examination

A 58‐year‐old male patient with a history of chronic lymphocytic leukemia (CLL) for the past 5 years arrives at the emergency room with symptoms of fever, productive cough, and shortness of breath (function class II dyspnea). The patient has been experiencing dyspnea for 1 month, and it has worsened over the past week. The patient reported experiencing a loss of appetite and a weight loss of approximately 2.5 kg over the past week (the patient's present weight was 71 kg), accompanied by night sweats and myalgia. The patient was a former cigarette smoker who quit 7 years ago. The physical examination revealed the following measurements: blood pressure of 115/80 mmHg, heart rate of 103 beats per minute, respiratory rate of 31 per minute, axillary temperature of 38.5°C, and oxygen saturation of 94%. Other systemic examination did not reveal any abnormality.

### 2.1 Past Medical History

Past medical history was positive for ischemic heart disease with patient underwent percutaneous coronary intervention 10 years ago. The patient was also a known case of CLL 5 years ago, received chemotherapy treatment with fludarabine, cyclophosphamide, and prednisolone. The patient was also taking folic acid 5 mg once a day and cotrimoxazole 480 mg once daily.

### 2.2 Differential Diagnosis

Identifying the underlying cause of ongoing dyspnea in individuals with hematologic malignancies can pose a considerable challenge. Typically, potential causes encompass a range of cardiovascular and non‐cardiovascular conditions, such as pneumonia, embolic events, anemia, or heart failure. In our specific case, pneumonia was suspected, prompting a differential diagnosis that involved considering other mentioned diagnoses as well.

### 2.3 Investigations

**FIGURE 1:**
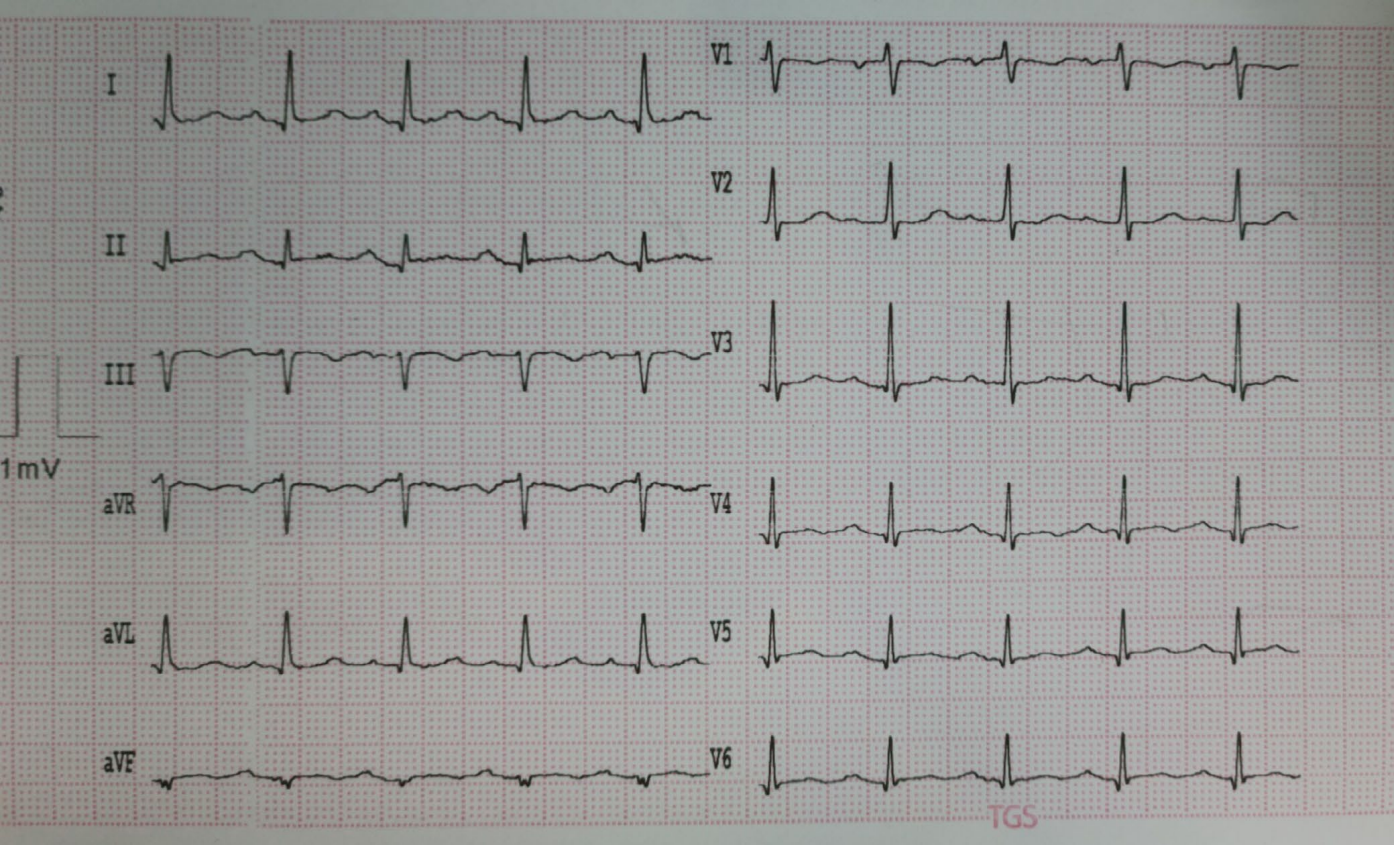
Electrocardiogram at presentation showing sinus tachycardia, normal sinus rhythm with nonspecific ST‐T changes.

**FIGURE 2:**
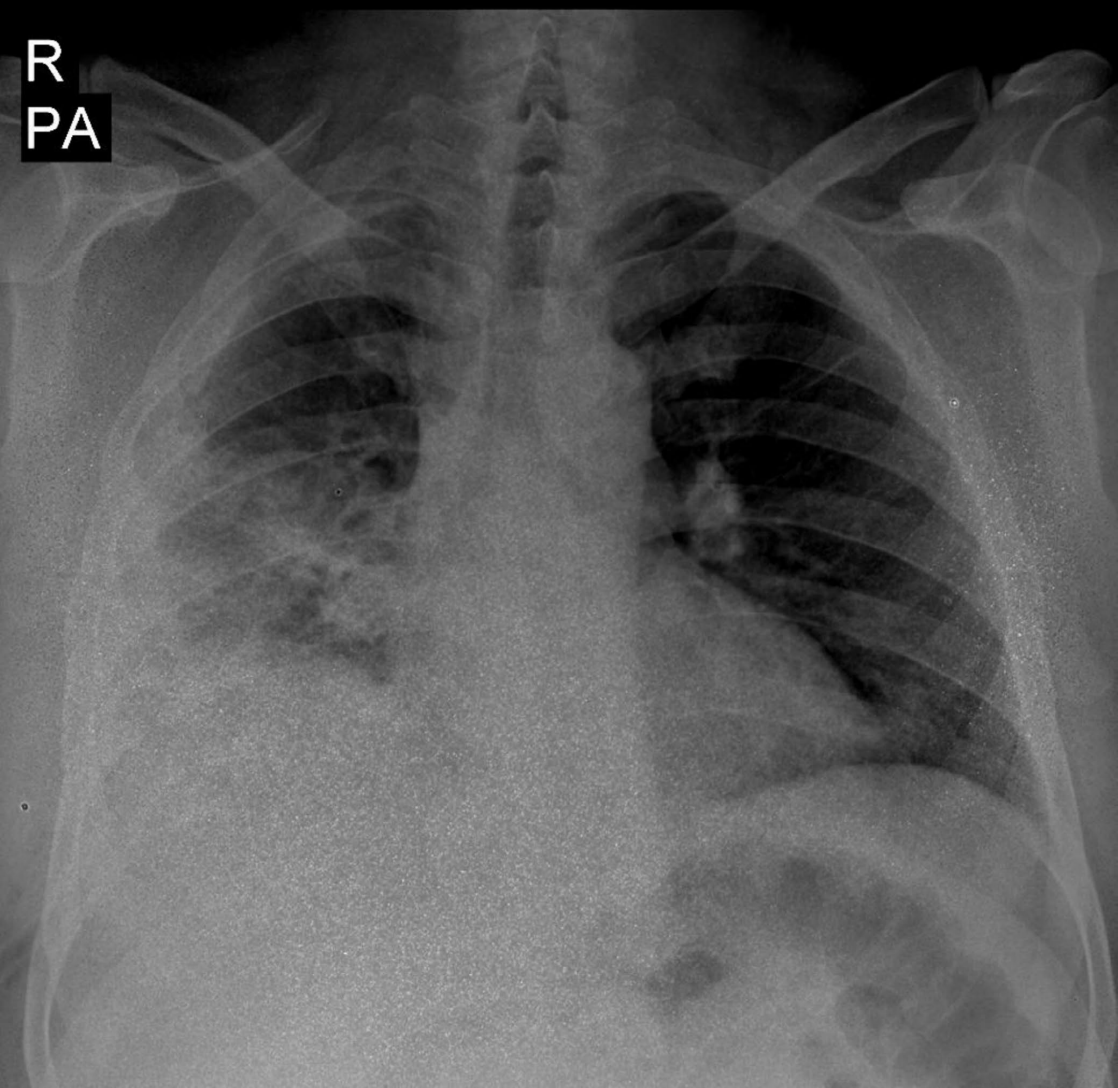
Chest x‐ray showed consolidations in lung fields, predominantly in right lung.

**FIGURE 3:**
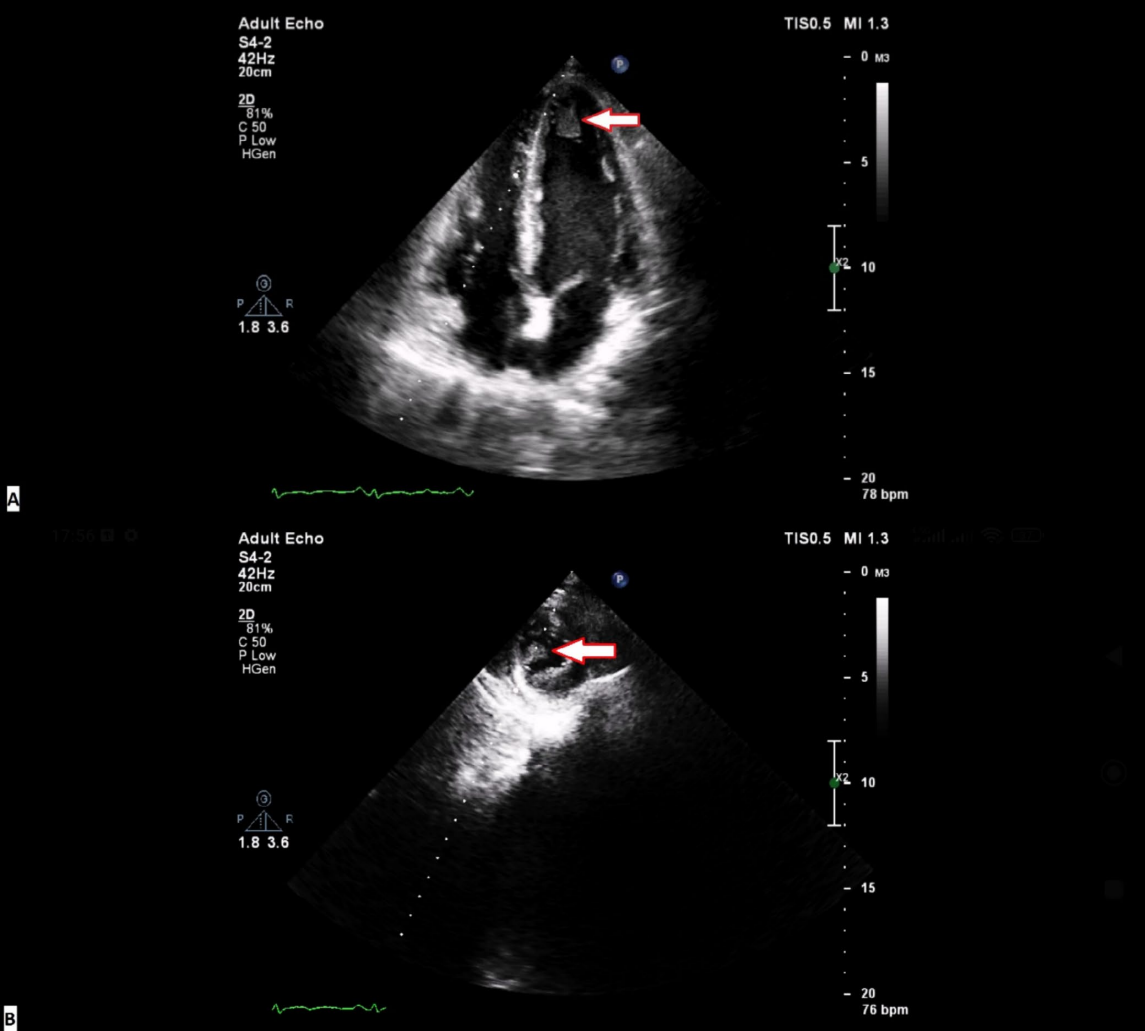
Left ventricular mass on transthoracic echocardiography. (A) Apical four‐chamber (A4C) view. (B) Left ventricular short axis view.

The complete blood count was significant for leukocytosis and mild anemia. The arterial blood gas test showed respiratory acidosis (pH: 7.17, PCO_2_: 72.4, and HCO_3_: 22.4). The remaining laboratory data of the patient can be seen in Table 1. An electrocardiogram was obtained from the patient, which revealed sinus tachycardia, a normal sinus rhythm with nonspecific ST‐T changes (Figure 1). The chest x‐ray of the patient revealed bilateral consolidation of the lower portions of the lungs, predominantly the right side, with loss of silhouette sign of heart borders (Figure 2). A TTE was performed on the patient according to their dyspnea over the past month. On echocardiography, a mobile large nonhomogenous mass with dimensions of 2.4 × 1 cm originating from the left ventricular (LV) apex with a narrow base and protruding into the LV cavity but without any obvious invasion of the myocardium was noted (Figure 3). Moreover, no regional wall motion abnormality (hypokinesia, akinesia) was reported (Videos 1 and 2).

**TABLE 1:** Patient's laboratory data on admission.

Test	Value	Unit	Normal range
WBC	25.4	*1000/μL	4.4–11.3
N%	30.5		
L%	64.3		
RBC	4.42	*10^6^/μL	4.5–5.1
Hb	11.8	g/dL	12.3–15.3
Hct	32.2	%	36–45
MCV	89.8	pg	26.5–32.5
MCH	28.4	fL	80–96
MCHC	33.4	g/dL	33–36
RDW‐CV	18.1	%	11.5–15
PLT	156	*1000/μL	150–450
MPV	11.1	fL	8.6–12.7
BS	100	mg/dL	
Calcium	8.0	mg/dL	8.5–10.5
Phosphorus	4.3	mg/dL	2.7–4.5
Magnesium	2.2	mg/dL	1.7–2.7
Albumin	3.7	g/dL	3.5–5.5
AST	44	u/L	5–40
ALT	23	u/L	5–40
ALP	173	u/L	< 258
LDH	839	u/L	< 480
Total bilirubin	0.7	mg/dL	< 1.1
Direct bilirubin	0.1	mg/dL	< 0.4
PT			
Patient	14.6	Seconds	11–13
Control	12.5	Seconds	
INR	1.2		0.8–1.2
APTT			
Control	26	Seconds	
Patient	30	Seconds	25–35
ABG			
pH	7.17		Arterial blood: 7.35–7.45
PO_2_	45.7	mmHg	Venous blood: 7.31
PCO_2_	72.4	mmHg	35–45
HCO_3_	22.4	mEq/L	22–26
CRP	50	mg/dL	0–8
Sodium	130	mEq/L	135–145
Potassium	4.9	mEq/L	3.5–5.3
ESR	44	mm/h	Men: < 15 mm/h Women: < 20 mm/h
Blood culture			
Culture 1	No growth after 24 h		
Culture 2	No growth after 72 h		

Abbreviations: ALP, alkalaine phosphatase; ALT, alanine aminotransferase; AST, aspartate aminotransferase; BS, blood sugar; CRP, C‐reactive protein; ESR, erythrocyte sedimentation rate; Hb, hemoglobin; INR, international normalized ratio; LDH, lactate dehydrogenase; MCH, mean corpuscular hemoglobin; MCHC, mean corpuscular hemoglobin concentration; MCV, mean corpuscular volume; PLT, platelet count; PT, prothrombin time; PTT, partial thromboplastin time; RDW‐CV, red cell distribution width—coefficient of variation; WBC, white blood cell count.

**VIDEO 1:** Left ventricular mass in apical four‐chamber view on echocardiography. Video content can be viewed at https://onlinelibrary.wiley.com/doi/10.1002/ccr3.70553

**VIDEO 2:** Left ventricular mass in left ventricular short‐axis view on echocardiography. Video content can be viewed at https://onlinelibrary.wiley.com/doi/10.1002/ccr3.70553

### 2.4 Management

Antibiotic therapy with cefepime and levofloxacin was initiated for the patient due to their clinical and paraclinical findings indicating pneumonia. Moreover, the patient was prescribed enoxaparin at a dosage of 1 mg/kg twice daily for the suspected diagnosis of a left ventricular thrombus according to the extremely rare incidence of primary cardiac tumors and infrequent occurrence of metastasis in CLL. A follow‐up echocardiography is scheduled in a month. After improvement in the general condition, the patient was discharged from the hospital with anticoagulation therapy of enoxaparin 60 mg daily once. Since the course of anticoagulation therapy could be punctuated by several issues like recurrence, hemorrhage, thrombocytopenia, new medications, conditions that affect oral intake or drug absorption, cancer progression, or achievement of remission, the patient was advised to visit every month to determine whether to continue or discontinue anticoagulation therapy. Following 1 month of anticoagulation treatment, subsequent echocardiography (Videos 3 and 4) showed no remaining signs of a left ventricular mass. Also, there was no sign of any embolic event in the patient, indicating the absence of embolization and migration of the suspected blood clot from its source. An ejection fraction (EF) of 55% and lack of apical wall motion abnormality were also appreciated in echocardiography. Hence, the anticoagulation therapy was discontinued.

**VIDEO 3:** Control echocardiography and resolution of left ventricular mass in A4C view. Video content can be viewed at https://onlinelibrary.wiley.com/doi/10.1002/ccr3.70553

**VIDEO 4:** Control echocardiography and resolution of left ventricular mass in left ventricular short‐axis view. Video content can be viewed at https://onlinelibrary.wiley.com/doi/10.1002/ccr3.70553

## 3 Discussion

The primary causes of intracardiac masses are thrombus, tumors, and vegetation [1]. For left ventricular masses, the differential diagnosis includes thrombus, primary tumors, and metastases, which must be distinguished from normal anatomical variations such as papillary muscles, false tendons, sigmoid septum, and apical trabeculations, which can be mistaken [2]. Thrombus is the most frequent cause of LV mass, commonly linked to coronary artery disease, LV aneurysm, cardiomyopathy, and myocarditis, and typically associated with wall motion abnormalities [2]. The primary concern with LV thrombus is the risk of thromboembolism, potentially leading to stroke, myocardial infarction (MI), or death [3]. A thrombus is described as an echo‐dense mass within the left ventricle that has an identifiable margin and is attached to the LV wall. It is accompanied by abnormal wall motion, either hypokinetic or akinetic [4, 5]. In this case, echocardiography reports no regional wall motion abnormality, which decreases the likelihood of left ventricular thrombus. The incidence of primary cardiac tumors is extremely rare [6] and the patient's previous echocardiography reports from the past year did not indicate any abnormalities related to cardiac tumors. Furthermore, there were no signs of wall invasion or pericardial effusion, which are typically associated with cardiac tumors rather than left ventricular thrombus [7]. Secondary tumors also seem infrequent as the patient's underlying malignancy was CLL. However, CLL does involve widespread distribution of cancerous cells, yet this is distinct from the metastatic process observed in solid tumors [8]. Therefore, the traditional concept of metastasis does not apply as frequently to CLL [9]. Furthermore, reports indicate that certain drugs in the patient's chemotherapy regimen, such as cyclophosphamide, are linked to hypercoagulability and thrombus formation via mechanisms including endothelial injury or protein C deficiency. This association enhances the probability of a thrombus‐related origin of the cardiac mass [10, 11, 12, 13, 14, 15]. Accordingly, based on the clinical intuitions in favor of the diagnosis of LV thrombus, administration of anticoagulation was commenced for the patient with monthly follow‐up. TTE with contrast or long inversion time late gadolinium enhancement cardiac magnetic resonance imaging can be used to monitor resolution of thrombus with anticoagulation [16, 17, 18]. Given the previous visualization of the thrombus on TTE, it was more reasonable and more convenient to obtain a follow‐up TTE rather than utilizing alternative diagnostic methods. After 1 month of anticoagulation therapy with enoxaparin, there was no remaining evidence of a left ventricular mass in control echocardiography. Generally, it is anticipated that a left ventricular thrombus will be resolved within a period of 3–6 months [18, 19]. Typically, when there is a noticeable improvement in the left ventricular ejection fraction and the abnormal motion of the apical wall is resolved, it is reasonable to consider reducing the duration of anticoagulation [19]. As these factors were reported to be normal on control echocardiography, and there was no evidence of any signs or symptoms suggestive of the embolization and movement of the suspected thrombus from the origin in the patient during the follow‐up and hospitalization, the resolution of left ventricular thrombus was confirmed, and the anticoagulation therapy was terminated. Overall, the case exhibits that even without suggestive echocardiographic features for thrombus in the left ventricle, like regional wall motion abnormality, individuals with a known malignancy are at a higher risk for developing thrombus due to a hypercoagulable state and increased likelihood of thrombotic complications. A more concerning scenario is that individuals with cancer may also have a higher risk of bleeding with anticoagulation. This complicates the decision‐making process regarding the use of anticoagulants in these patients. Therefore, commencing anticoagulant therapy for these patients necessitates a thorough assessment of the patient, as well as a careful consideration of the bleeding risk and the advantages of anticoagulation therapy. In this case, after control echocardiography indicated no remaining sign of LV thrombus, and an EF of 55%, the anticoagulation therapy was ceased in order to prevent the development of adverse effects or condition.

Additionally, it is pertinent to note in this context that patients with hematological malignancies are at increased risk for cardiovascular complications, influenced by the disease type and stage, along with patient‐specific and therapy‐related risk factors that negatively affect survival outcomes [20]. A thorough cardiac evaluation prior to, during, and following treatment is essential. The integration of surveillance strategies facilitates early detection and management, ultimately enhancing survival rates through the collaborative efforts of hematologists and cardio‐oncologists to ensure optimal medical care.

## 4 Conclusions

Left ventricular thrombus is commonly found in patients with anterior ST‐elevation MI and anteroapical infarcts, where there are large areas of poorly contracting left ventricular muscle. This can be observed on echocardiography as a regional wall motion abnormality. Additionally, patients with hypercoagulable conditions such as malignancies are also at risk for developing left ventricular thrombus and the diagnosis is challenging because the echocardiographic features of LV mass could be less compatible with the LV thrombus.

## 5 Take Home Messages

1. To be able to make a differential diagnosis of left ventricular mass on echocardiography.
2. To understand the probability of left ventricular clot as an incidental mass on echocardiography even in the absence of abnormal regional wall motion.
3. Echocardiography may be highly beneficial in the clinical management of various hematologic malignancies and potentially other conditions.

## Author Contributions

**Hedieh Alimi:** conceptualization, methodology, project administration, resources, supervision, visualization. **Ali Tajik:** conceptualization, data curation, supervision, writing – original draft, writing – review and editing.

## Consent

Written informed consent was obtained from the patient for publication of this case report and accompanying images.

## Conflicts of Interest

The authors declare no conflicts of interest.

## Data Availability

The data used to support the findings of this study are included in the article.
